# 3D-dynamic representation of DNA sequences

**DOI:** 10.1007/s00894-014-2141-8

**Published:** 2014-02-25

**Authors:** Piotr Wąż, Dorota Bielińska-Wąż

**Affiliations:** 1 Department of Nuclear Medicine, Medical University of Gdańsk, Tuwima 15, 80-210 Gdańsk, Poland; 2 Department of Radiological Informatics and Statistics, Medical University of Gdańsk, Tuwima 15, 80-210 Gdańsk, Poland

**Keywords:** Descriptors, DNA sequences, Moments of inertia

## Abstract

A new 3D graphical representation of DNA sequences is introduced. This representation is called 3D-dynamic representation. It is a generalization of the 2D-dynamic dynamic representation. The sequences are represented by sets of “material points” in the 3D space. The resulting 3D-dynamic graphs are treated as rigid bodies. The descriptors characterizing the graphs are analogous to the ones used in the classical dynamics. The classification diagrams derived from this representation are presented and discussed. Due to the third dimension, “the history of the graph” can be recognized graphically because the 3D-dynamic graph does not overlap with itself. Specific parts of the graphs correspond to specific parts of the sequence. This feature is essential for graphical comparisons of the sequences. Numerically, both 2D and 3D approaches are of high quality. In particular, a difference in a single base between two sequences can be identified and correctly described (one can identify which base) by both 2D and 3D methods.

## Introduction

In modern biomedical sciences methods derived from physics, mathematics, and numerical analysis are frequently applied. Therefore this branch of science is, in fact, interdisciplinary. In particular, the analysis of biological sequences (DNA, RNA, protein) combines interdisciplinary methodology. Powerful methods are graphical representations which allow for both graphical and numerical characterization of the sequences. The sequences are usually very long, and it is not obvious how to represent these objects. The questions how to avoid the degeneracy and how to express the features of the objects both graphically and numerically, result in numerous methods.

In the present work, we introduce a new 3D graphical representation method. The proposed method is a 3D generalization of the 2D-dynamic representation of DNA sequences [1]. The 2D-dynamic graphs represent the DNA sequences. They are composed of the “material points” distributed in a 2D-space. Their distribution is determined by the sequence. We proposed the moments of inertia and the coordinates of the centers of mass of the 2D-dynamic graphs for the numerical characterization of the DNA sequences [1]. We also considered the high-order moments of the mass-density distributions based on 2D-dynamic graphs as the descriptors [2]. The mass overlaps and the angles between X axis and the principal axis of inertia are also used for the description of similarity/dissimilarity of the DNA sequences [3].

Both our methods (2D and 3D-dynamic representations) are based on a walk in a space which is one of the common approaches in this field. The 2D graphical representation methods took their origin in visualizations of these walks [4–6]. The approaches based on a walk in a 3D space may be found in [7–11]. The differences between them are due to assigning different basis vectors to particular bases and due to different numerical characterizations of the graphs. Examples of various 3D graphical representation methods may be found in [12–23].

In the present work we model a DNA sequence as a set of “material points” in the 3D space. As a consequence, the sequence is characterized by the dynamical quantities, e.g., moments of inertia, analogously as in 2D-dynamic representations. Therefore we retained the name ‘3D-dynamic representation of DNA sequences’. Using the new model we construct the classification diagrams.

## Method

The proposed method is based on the convention of a walk in a 3D space. A base in a sequence is represented by a material point in the 3D space. To each point an abstract mass is assigned. We start the walk in the point with coordinates (0,0). In each step this point is shifted by a unit vector. We represent the bases by the following unit vectors: A = (−1,0,1), G = (1,0,1), C = (0,1,1), and T = (0,−1,1). At the end of the vector we locate a mass *m* = 1. As a consequence, the 3D-dynamic graph is obtained. It consists of the material points in the 3D space with the unit masses. The distribution of the points in the space is determined by the sequence.

The coordinates of the center of mass of the 3D-dynamic graph, in the {*X*,*Y*,*Z*} coordinate system are defined as

1 $$ {\mu}_x=\frac{{\displaystyle {\sum}_i}\;{m}_i{x}_i}{{\displaystyle {\sum}_i}\;{m}_i},\kern2em {\mu}_y=\frac{{\displaystyle {\sum}_i}\;{m}_i{y}_i}{{\displaystyle {\sum}_i}\;{m}_i},\kern2em {\mu}_z=\frac{{\displaystyle {\sum}_i}\;{m}_i{z}_i}{{\displaystyle {\sum}_i}\;{m}_i}, $$

where *x*_*i*_, *y*_*i*_, *z*_*i*_ are the coordinates of the mass *m*_*i*_. Since *m*_*i*_ = 1 for all the points, the total mass of the sequence is *N* = ∑ _*i*_ *m*_*i*_, where *N* is the length of the sequence. Then, the coordinates of the center of mass of the 3D-dynamic graph may be expressed as

2 $$ {\mu}_x=\frac{1}{N}{\displaystyle \sum_i}\;{x}_i,\kern2em {\mu}_y=\frac{1}{N}{\displaystyle \sum_i}\;{y}_i,\kern2em {\mu}_z=\frac{1}{N}{\displaystyle \sum_i}\;{z}_i. $$

The tensor of the moment of inertia is given by the matrix

3 $$ \widehat{I}=\left(\begin{array}{ccc}\hfill {I}_{xx}\hfill & \hfill {I}_{xy}\hfill & \hfill {I}_{xz}\hfill \\ {}\hfill {I}_{yx}\hfill & \hfill {I}_{yy}\hfill & \hfill {I}_{yz}\hfill \\ {}\hfill {I}_{zx}\hfill & \hfill {I}_{zy}\hfill & \hfill {I}_{zz}\hfill \end{array}\right) $$

with

4 $$ \begin{array}{c}\hfill {I}_{xx}={\displaystyle \sum_i}\;{m}_i\left[{\left({y}_i^{\mu}\right)}^2+{\left({z}_i^{\mu}\right)}^2\right],\hfill \\ {}\hfill {I}_{yy}={\displaystyle \sum_i}\;{m}_i\left[{\left({x}_i^{\mu}\right)}^2+{\left({z}_i^{\mu}\right)}^2\right],\hfill \\ {}\hfill {I}_{zz}={\displaystyle \sum_i}\;{m}_i\left[{\left({x}_i^{\mu}\right)}^2+{\left({y}_i^{\mu}\right)}^2\right],\hfill \\ {}\hfill {I}_{xy}={I}_{yx}=-{\displaystyle \sum_i}\;{m}_i{x}_i^{\mu }{y}_i^{\mu },\hfill \\ {}\hfill {I}_{xz}={I}_{zx}=-{\displaystyle \sum_i}\;{m}_i{x}_i^{\mu }{z}_i^{\mu },\hfill \\ {}\hfill {I}_{yz}={I}_{zy}=-{\displaystyle \sum_i}\;{m}_i{y}_i^{\mu }{z}_i^{\mu },\hfill \end{array} $$

where *x*_*i*_^*μ*^, *y*_*i*_^*μ*^, *z*_*i*_^*μ*^ are the coordinates of *m*_*i*_ in the Cartesian coordinate system for which the origin has been selected at the center of mass.

The eigenvalue problem of the tensor of inertia is defined as

5 $$ \widehat{I}{\omega}_k={I}_k{\omega}_k,\kern1.5em k=1,2,3, $$

where *I*_*k*_ are the eigenvalues and *ω*_*k*_–the eigenvectors. The eigenvalues are obtained by solving the third-order secular equation

6 $$ \left|\begin{array}{ccccc}\hfill {I}_{xx}-I\hfill & \hfill \hfill & \hfill {I}_{xy}\hfill & \hfill \hfill & \hfill {I}_{xz}\hfill \\ {}\hfill {I}_{yx}\hfill & \hfill \hfill & \hfill {I}_{yy}-I\hfill & \hfill \hfill & \hfill {I}_{yz}\hfill \\ {}\hfill {I}_{zx}\hfill & \hfill \hfill & \hfill {I}_{zy}\hfill & \hfill \hfill & \hfill {I}_{zz}-I\hfill \end{array}\right|=0. $$

The eigenvectors *ω*_1_, *ω*_2_, *ω*_3_ are orthonormal. Thus, they form a basis for a new coordinate system. The corresponding axes of this new system are denoted *Ω*_1_, *Ω*_2_, *Ω*_3_ and referred to as the principal axes. The eigenvalues *I*_1_, *I*_2_, *I*_3_, are called the principal moments of inertia and are equal to the moments of inertia associated with the rotations around the principal axes.

The relative orientation of the new and old coordinate system may be described by the cosines of properly defined angles. Let *M*_1_, *M*_2_, and *M*_3_ denote, respectively, the planes (*X*,*Y*), (*X*,*Z*), and (*Y*,*Z*). Similarly, *N*_1_, *N*_2_, *N*_3_ stand for the planes (*Ω*_1_,*Ω*_2_), (*Ω*_1_,*Ω*_3_), (*Ω*_2_,*Ω*_3_), respectively. For the characterization of the 3D-dynamic graphs we use the cosines of the angles between the planes of the two systems of coordinates:

7 $$ {C}_{ij}\equiv cos\left({M}_i,{N}_j\right),\kern1.5em i,j=1,2,3. $$

It is also convenient to use square roots of the normalized principal moments of inertia:

8 $$ {r}_1=\sqrt{\frac{I_1}{N}},\kern2em {r}_2=\sqrt{\frac{I_2}{N}},\kern2em {r}_3=\sqrt{\frac{I_3}{N}}. $$

As the descriptors of the 3D-dynamic graphs we take:

- The coordinates of the centers of mass of the graphs,
- The principal moments of inertia of the graphs,
- The values of *C*_*ij*_.

## Results and discussion

The new approach has been applied to histone H4 coding sequences of different species listed in Table 1 and for alpha globin coding sequences of different species listed in Table 4. The lengths of all histone H4 coding sequences are *N* = 312 and of all alpha globing coding sequences are *N* = 429.

**Table 1:** Coordinates of the centers of mass of the graphs representing histone H4 coding sequences

No.	Species	Gene ID (EMBL)	*μ*_*x*_	*μ*_*y*_	*μ*_*z*_
1	chicken	M74533	26.95	34.15	156.5
2	chicken	M74534	26.95	34.29	156.5
3	human	M60749	12.34	9.228	156.5
4	mouse	V00753	17.86	19.25	156.5
5	rat	M27433	16.92	17.93	156.5
6	wheat	M12277	24.93	34.84	156.5
7	maize	M36659	28.59	25.55	156.5
8	maize	M13370	29.22	27.84	156.5
9	maize	M13377	29.48	25.68	156.5

**Fig. 1:**
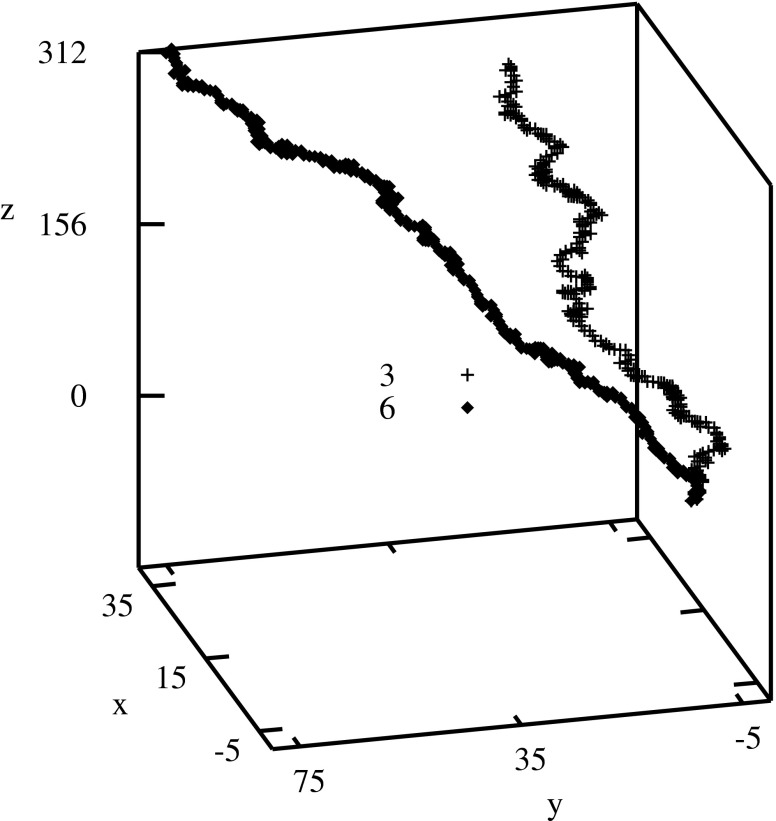
Examples of 3D-dynamic graphs: No. 3 (M60749, former gene ID HSHISAD) and 6: (M12277, former gene ID TAH4091)–see Table 1

Some examples of 3D-dynamic graphs are shown in Fig. 1.

**Fig. 2:**
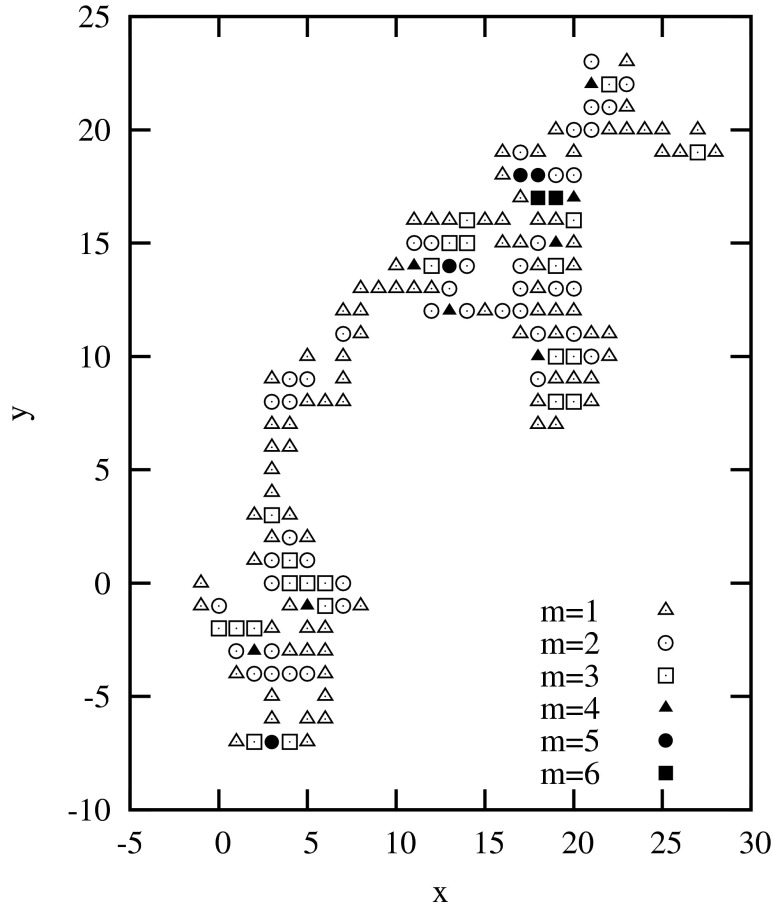
2D-dynamic graph: No. 3 (M60749)

Figure 2 shows 2D-dynamic graph for the same sequence (No. 3 in Table 1) as in Fig. 1. 2D-dynamic graphs remove the degeneracy present in the Nandy plots [5]. This degeneracy comes from the so called repetitive walks (walks performed back and forth along the same trace). By the introduction in the 2D-dynamic graphs points with different masses the repetitive walks can be recognized both graphically and numerically (the descriptors depend on masses different than 1). However, the 2D-dynamic graphs still do not retain the history of the sequence. Introducing the third dimension one can avoid self-overlapping of the graph.

Numerically, each graph is characterized by descriptors. The values of the descriptors considered in this work are shown in Tables 1, 2, 3, 4, 5, and 6. Due to the choice of the unit vectors representing the four bases, *μ*_*x*_ and *μ*_*y*_ give information about the relative number of particular bases in the sequences, and *μ*_*z*_ contains information about the lengths of the sequences only. *μ*_*x*_ and *μ*_*y*_ shown in Tables 1 and 4 are identical to *μ*_*x*_ and *μ*_*y*_ for the 2D-dynamic graphs for the same sequences [1]. New information is contained in other descriptors (Tables 2, 3, 5, and 6). The descriptors are very sensitive: they correctly identify a single-base difference between two sequences. The sequence no. 6 in Table 4 (EF605407) differs by two bases from the sequence (MMAGL1) used in the calculations in [1]. The base T in MMAGL1 is replaced by C in EF605407 on the 132 position in the sequence, and the base A in MMAGL1 is replaced by G in EF605407 on the 366 position in the sequence. As a consequence of the change T to C *μ*_*y*_ increased, and as a consequence of the change A to G *μ*_*x*_ increased: *μ*_*x*_ = 15.49, *μ*_*y*_ = 14.80 for MMAGL1, and *μ*_*x*_ = 15.79, *μ*_*y*_ = 16.19 for EF605407.

**Table 2:** Principal moments of inertia of the graphs and cosines of the angles relative to *M*_1_ representing histone H4 coding sequences

No.	*I*_1_	*I*_2_	*I*_3_	*C*_11_	*C*_12_	*C*_13_
1	2718517.5	2717050.5	5248.7777	0.9654	−0.2012	0.1657
2	2721386.8	2719932.1	5123.5590	0.9649	−0.1860	0.1854
3	2567018.1	2569325.3	5702.8901	0.9933	−0.0931	0.0690
4	2629277.7	2630996.7	4799.7393	0.9814	−0.1898	0.0291
5	2641789.4	2644023.6	5243.5711	0.9791	−0.1611	0.1245
6	2718890.3	2723552.5	6553.1238	0.9650	−0.2624	−0.0018
7	2657698.2	2660580.3	4894.8295	0.9760	−0.2120	−0.0495
8	2677850.1	2681309.5	6696.9065	0.9725	−0.2323	0.0186
9	2652990.0	2655433.3	5383.5528	0.9770	−0.1951	−0.0864

**Table 3:** Cosines of the angles relative to *M*_2_ and *M*_3_ representing histone H4 coding sequences

No.	*C*_21_	*C*_22_	*C*_23_	*C*_31_	*C*_32_	*C*_33_
1	0.2222	0.3029	−0.9268	0.1363	0.9315	0.3371
2	0.2245	0.2180	−0.9498	0.1362	0.9581	0.2521
3	0.0849	0.1791	−0.9802	0.0789	0.9794	0.1858
4	0.1609	0.7302	−0.6640	0.1048	0.6563	0.7472
5	0.1767	0.3678	−0.9130	0.1013	0.9158	0.3886
6	0.2422	0.8932	−0.3788	0.1010	0.3651	0.9255
7	0.1781	0.9085	−0.3780	0.1251	0.3601	0.9245
8	0.1920	0.7530	−0.6293	0.1322	0.6156	0.7769
9	0.1713	0.9587	−0.2271	0.1271	0.2071	0.9700

**Table 4:** Coordinates of the centers of mass of the graphs representing alpha globing coding sequences

No.	Species	Gene ID (EMBL)	*μ*_*x*_	*μ*_*y*_	*μ*_*z*_
1	goat	EU938069	26.01	33.03	215.0
2	chicken	M15379	2.312	33.62	215.0
3	rhesus monkey	J04495	31.01	36.42	215.0
4	orangutan	M12157	23.43	40.97	215.0
5	horse	M17902	23.02	38.12	215.0
6	mouse	EF605407	15.79	16.19	215.0
7	rabbit	M11113	12.94	36.67	215.0

**Table 5:** Principal moments of inertia of the graphs and cosines of the angles relative to *M*_1_ representing alpha globing coding sequences

No.	*I*_1_	*I*_2_	*I*_3_	*C*_11_	*C*_12_	*C*_13_
1	6868772.7	6870362.4	7983.8311	0.9789	−0.1979	0.0503
2	6788337.0	6796077.5	11107.903	0.9846	−0.1694	0.0428
3	6975843.0	6978180.6	7307.5904	0.9713	−0.2362	0.0266
4	6948275.6	6949747.9	5325.5281	0.9732	−0.2283	−0.0271
5	6893025.9	6894510.4	7514.3009	0.9772	−0.1935	−0.0875
6	6730034.2	6726610.9	9920.4895	0.9892	−0.1446	−0.0226
7	6886040.9	6887794.4	7488.2889	0.9777	−0.2058	0.0425

**Table 6:** Cosines of the angles relative to *M*_2_ and *M*_3_ representing alpha globing coding sequences

No.	*C*_21_	*C*_22_	*C*_23_	*C*_31_	*C*_32_	*C*_33_
1	0.1779	0.7052	−0.6863	0.1003	0.6808	0.7256
2	0.1728	0.9075	−0.3828	0.0260	0.3843	0.9228
3	0.1989	0.7466	−0.6348	0.1301	0.6219	0.7722
4	0.2076	0.9233	−0.3231	0.0988	0.3088	0.9460
5	0.1931	0.9811	−0.0133	0.0885	−0.0039	0.9961
6	0.1207	0.8933	−0.4329	0.0828	0.4255	0.9012
7	0.2001	0.8502	−0.4870	0.0641	0.4846	0.8724

**Fig. 3:**
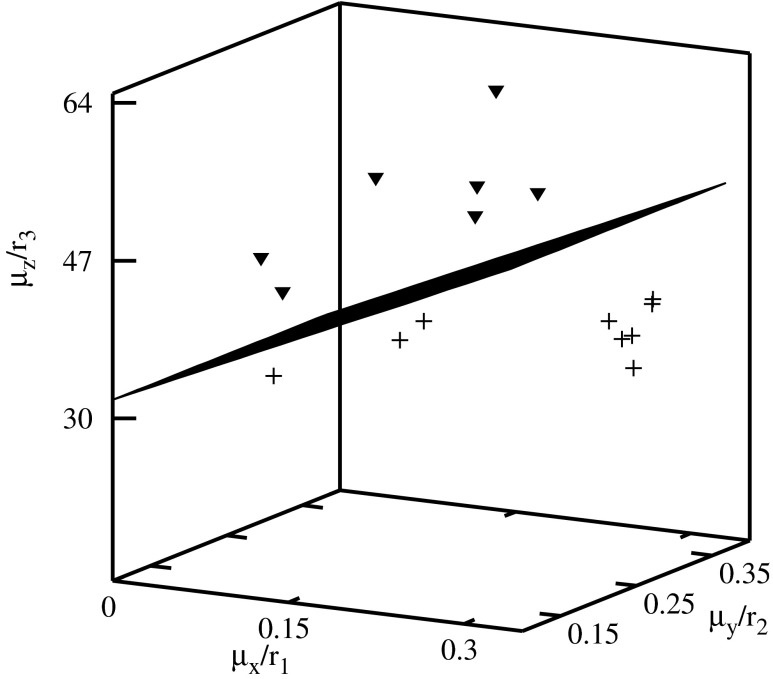
Classification diagram $$ {\scriptscriptstyle \frac{\mu_x}{r_1}} $$–$$ {\scriptscriptstyle \frac{\mu_y}{r_2}} $$–$$ {\scriptscriptstyle \frac{\mu_z}{r_3}} $$

**Fig. 4:**
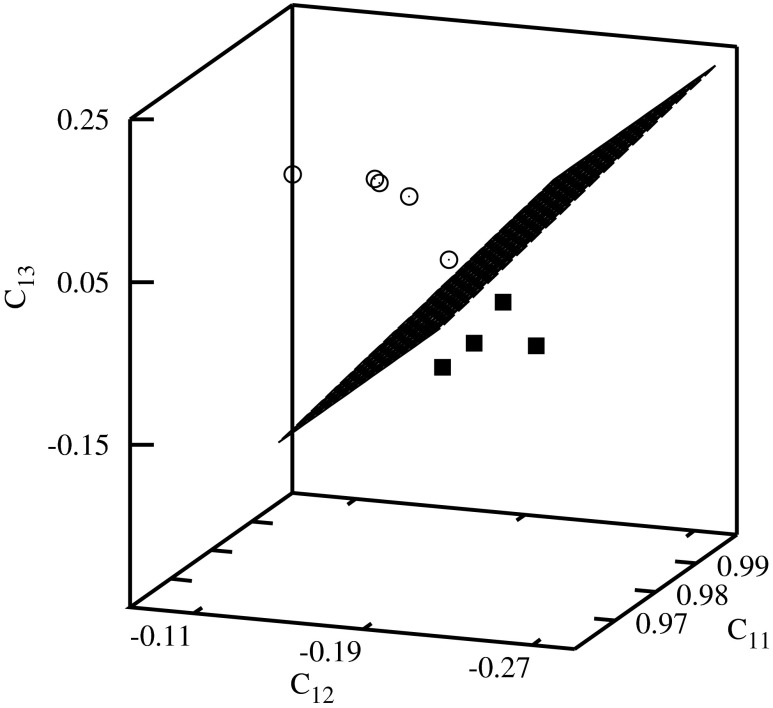
Classification diagram *C*_11_–*C*_12_–*C*_13_

**Fig. 5:**
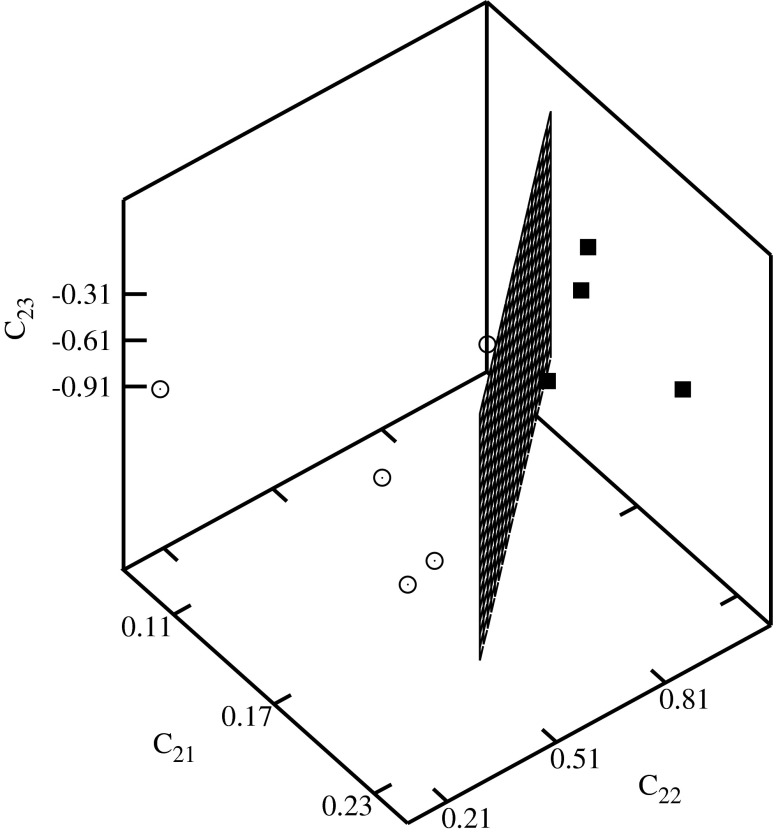
Classification diagram *C*_21_–*C*_22_–*C*_23_

**Fig. 6:**
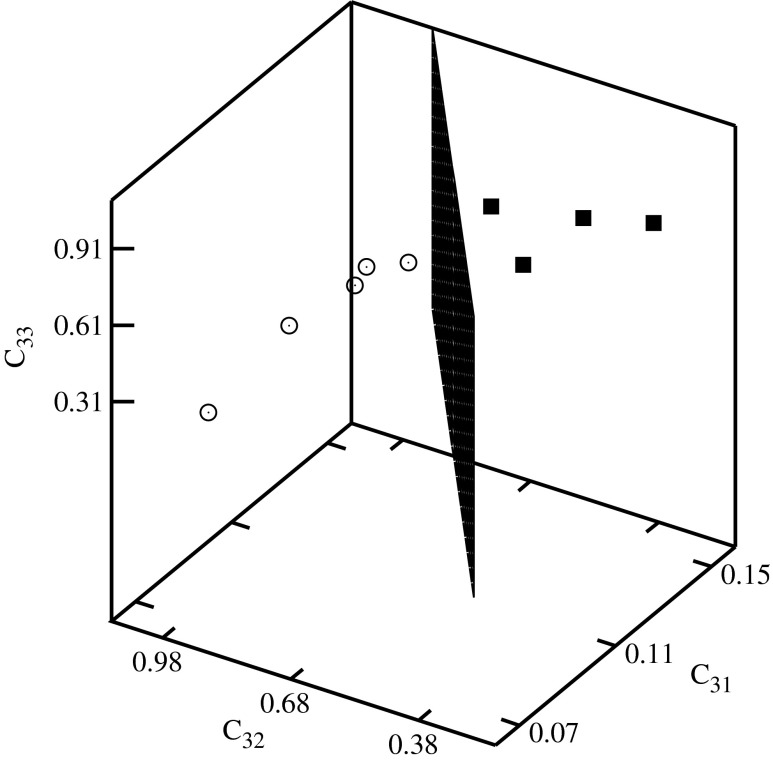
Classification diagram *C*_31_–*C*_32_–*C*_33_

**Fig. 7:**
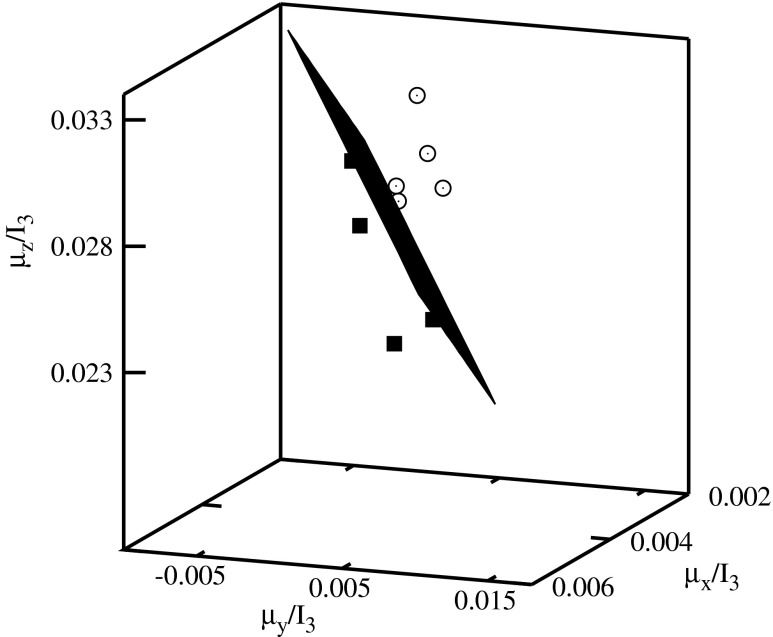
Classification diagram $$ {\scriptscriptstyle \frac{\mu_x}{I_3}} $$–$$ {\scriptscriptstyle \frac{\mu_y}{I_3}} $$–$$ {\scriptscriptstyle \frac{\mu_z}{I_3}} $$

**Fig. 8:**
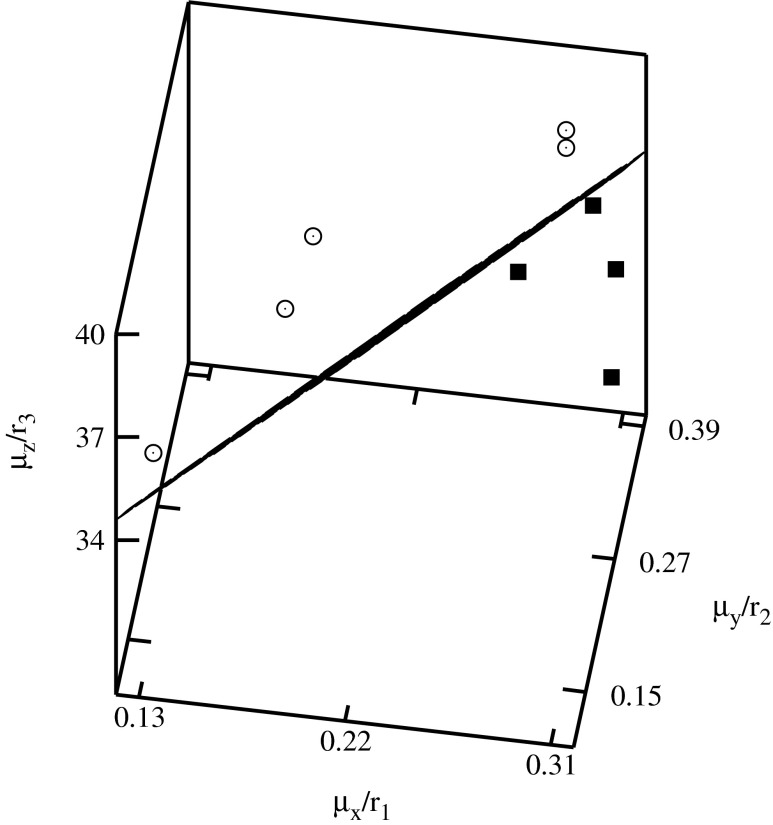
Classification diagram $$ {\scriptscriptstyle \frac{\mu_x}{r_1}} $$–$$ {\scriptscriptstyle \frac{\mu_y}{r_2}} $$–$$ {\scriptscriptstyle \frac{\mu_z}{r_3}} $$

The descriptors have been used for the construction of the classification diagrams shown in Figs. 3, 4, 5, 6, 7, and 8. Figure 3 shows the classification diagram ${\scriptscriptstyle \frac{\mu_x}{r_1}}$–${\scriptscriptstyle \frac{\mu_y}{r_2}}$–${\scriptscriptstyle \frac{\mu_z}{r_3}}$. The descriptors representing histone H4 coding sequences are represented in the figure by crosses and alpha globin coding sequences by triangles. The crosses and the triangles are located in a different part of the diagram. In the figure these parts are separated by a plane.

Using the present approach one can also create very detailed classification diagrams (in this case, for histone H4 coding sequences of evolutionary similar organisms). The similarity matrix using the standard Clustal W approach for histone H4 coding sequences we gave in [3] (the similarity values are either larger or equal 78*%*). The considered sequences are rather similar to each other and it is difficult to find a property which allows to distinguish between different species. In particular a good test of the new methods is finding descriptors for which we observe clusterization of the descriptors representing sequences of evolutionarily similar organisms: plants and vertebrates for histone H4 coding sequences. Most of the descriptors give larger similarity values between the sequences of chicken (No. 1, 2 in Table 1) with the sequences of plants rather than with the ones of vertebrates. Using 2D-dynamic representation we found some properties that in effect give the classification of the sequences representing plants and vertebrates [24]. In the present work, we find more descriptors that give a similar classification.

The histone H4 coding sequences of plants are represented by the full squares, and of vertebrates by the empty circles in Figs. 4, 5, 6, 7, and 8. A clusterization of the sequences representing evolutionarily similar organisms is obtained for *C*_*ij*_,  *i*, *j* = 1, 2, 3 parameters (Figs. 4, 5, and 6) and for the descriptors composed of moments of inertia, coordinates of centers of mass of the graphs, and the coefficients *r*_*i*_,  *i* = 1, 2, 3 (Figs. 7 and 8). Figure 4 corresponds to *i* = 1, *j* = 1, 2, 3, Fig. 5 to *i* = 2, *j* = 1, 2, 3, and Fig. 6 to *i* = 3, *j* = 1, 2, 3.

The descriptors representing the sequences of plants and of vertebrates are located in different parts of the diagrams. In order to visualize the classifications, the clusters of descriptors corresponding to different species have been separated by planes.

Summarizing, both approaches (2D and 3D-dynamic representations) are examples of graphical representation methods. Very popular methods based on the alignment of the sequences give rather limited information about similarity/dissimilarity of the sequences. Their degeneracy is relatively high. The same similarity values are obtained if T, C, G, or A bases align. Using graphical representation methods one has a chance to consider different aspects of similarity separately, both graphically and numerically. The computing time of these methods is low.

The 3D-dynamic graphs are generalizations of the 2D-dynamic graphs. The descriptors used for the characterization of the graphs are also related to the dynamics. The proposed descriptors of the 3D-dynamic graphs lead to new classifications diagrams for the considered data, analogously as for the 2D-dynamic graphs [24]. Therefore the descriptors proposed for both 2D and 3D-dynamic graphs are good, reliable and sensitive, tools for similarity/dissimilarity analysis of DNA sequences. The 3D-dynamic graphs retain the history of the sequences and this is one of their advantages. The consecutive bases in the sequences are represented by the appropriate parts of the 3D-dynamic graphs (the 3D graph never overlaps with itself). Therefore the future applications of the 3D method both as a graphical and as a numerical tool seem to be promising.
